# Supplementary material for: Addressing food insecurity among U.S. refugees, considering the temporal patterns of food insecurity after resettlement: Qualitative insights from Utah
Source: PLoS One. 2025 Jul 17;20(7):e0327645. doi: 10.1371/journal.pone.0327645 (PMC12270138; doi:10.1371/journal.pone.0327645)
Supplement: S1 Table — (DOCX) [file pone.0327645.s001.docx]

Title: Addressing food insecurity among U.S. refugees, considering the temporal patterns of food insecurity after resettlement: Qualitative insights from Utah.

# Supporting Material 1 - Consolidated Criteria for Reporting Qualitative Research (COREQ): 32-Item Checklist

Developed from: Tong A, Sainsbury P, Craig J. Consolidated criteria for reporting qualitative research (COREQ): a 32-item checklist for interviews and focus groups. *International journal for quality in health care*. 2007;19(6):349-357

S1 Table: The 32-item checklist based on the Consolidated Criteria for Reporting Qualitative Research (COREQ)

| No. Item | Guide questions/description | Reported on Page # |
| --- | --- | --- |
| Domain 1: Research team and reﬂexivity |  |  |
| *Personal Characteristics* | | |
| 1. Interviewer/facilitator | Which authors conducted the interview? | Page 5 – Three bilingual staff members conducted the interviews. They are not listed as authors but are mentioned in the Acknowledgement section. |
| 2. Credentials | What were the researcher’s credentials? | Page 1 – See the Title Page |
| 3. Occupation | What was their occupation at the time of the study? | Page 1 – See the Title Page |
| 4. Gender | Was the researcher male or female? | Page 6 – Data Analysis section |
| 5. Experience and training | What experience or training did the researcher have? | Page 6 – Data Analysis section |
| *Relationship with participants* |  |  |
| 6. Relationship established | Was a relationship established prior to study commencement? | Only with the resettlement agency. |
| 7. Participant knowledge of the interviewer | What did the participants know about the researcher? | Page 5 – Recruitment section |
| 8. Interviewer characteristics | What characteristics were reported about the interviewer? e.g. Bias, assumptions, reasons and interests in the research topic | We only let them know that the interviewers are female and the interview will take place at their home. |
| Domain 2: study design |  |  |
| Theoretical framework | | |
| 9. Methodological orientation and Theory | What methodological orientation was stated to underpin the study? e.g. grounded theory, discourse analysis, ethnography, phenomenology, content analysis | Page 6 – Data Analysis section |
| Participant selection | | |
| 10. Sampling | How were participants selected? e.g. purposive, convenience, consecutive, snowball | Page 5 – Recruitment section |
| 11. Method of approach | How were participants approached? e.g. face-to-face, telephone, mail, email | Page 5 – Recruitment section |
| 12. Sample size | How many participants were in the study? | Page 5 – Data Collection section |
| 13. Non-participation | How many people refused to participate or dropped out? Reasons? | Page 7 – Results section |
| Setting | | |
| 14. Setting of data collection | Where was the data collected? e.g. home, clinic, workplace | Page 5 – Data Collection section |
| 15. Presence of non-participants | Was anyone else present besides the participants and researchers? | Page 5 – Data Collection section |
| 16. Description of sample | What are the important characteristics of the sample? e.g. demographic data, date | Pages 7-8 – Results section |
| Data collection | | |
| 17. Interview guide | Were questions, prompts, guides provided by the authors? Was it pilot tested? | It is available upon request. |
| 18. Repeat interviews | Were repeat interviews carried out? If yes, how many? | No. |
| 19. Audio/visual recording | Did the research use audio or visual recording to collect the data? | Audio recording. |
| 20. Field notes | Were ﬁeld notes made during and/or after the interview? | No. |
| 21. Duration | What was the duration of the interviews? | Page 5 – Data Collection section |
| 22. Data saturation | Was data saturation discussed? | Pages 6-7 – Data Analysis section |
| 23. Transcripts returned | Were transcripts returned to participants for comment and/or correction? | No. |
| Domain 3: analysis and ﬁndings |  |  |
| Data analysis | | |
| 24. Number of data coders | How many data coders coded the data? | Page 6 – Data Analysis section |
| 25. Description of the coding tree | Did authors provide a description of the coding tree? | No. |
| 26. Derivation of themes | Were themes identiﬁed in advance or derived from the data? | From the data. |
| 27. Software | What software, if applicable, was used to manage the data? | Page 6-7 – Data Analysis section. Dedoose |
| 28. Participant checking | Did participants provide feedback on the ﬁndings? | No. |
| Reporting | | |
| 29. Quotations presented | Were participant quotations presented to illustrate the themes/ﬁndings? Was each quotation identiﬁed? e.g. participant number | Pages 9-15 and the Appendix |
| 30. Data and ﬁndings consistent | Was there consistency between the data presented and the ﬁndings? | Yes. |
| 31. Clarity of major themes | Were major themes clearly presented in the ﬁndings? | Yes. Pages 9-15 |
| 32. Clarity of minor themes | Is there a description of diverse cases or discussion of minor themes? | Yes. Notes of Table 2. |
