# Supplementary material for: Addressing food insecurity among U.S. refugees, considering the temporal patterns of food insecurity after resettlement: Qualitative insights from Utah
Source: PLoS One. 2025 Jul 17;20(7):e0327645. doi: 10.1371/journal.pone.0327645 (PMC12270138; doi:10.1371/journal.pone.0327645)
Supplement: S2 Table — (DOCX) [file pone.0327645.s002.docx]

Title: Addressing food insecurity among U.S. refugees, considering the temporal patterns of food insecurity after resettlement: Qualitative insights from Utah.

# Supporting Material 2 – Additional quotes related to each theme

S2 Table: Additional quotes related to themes from the main text

| Theme | Description | Quotes |
| --- | --- | --- |
| Themes related to food insecurity experiences | | |
| Affordability | The theme of Affordability reflects income-related barriers including the cost of food, the inability to afford food, unemployment due to limited opportunities or language barriers, and ineligibility for federal nutrition assistance programs such as SNAP for individuals whose income slightly exceeds program thresholds | We went to ask why they closed food stamp. And they called them and sent a paper saying that they closed it because the money we are making is enough (#46, Kinyarwanda, Female).  Food stamps should not be cut off, it is an essential thing. Here we don’t have an income, if you work you will live, if you don’t work you will be homeless. Okay we will work and pay our rent and our bills, but in my opinion a basic essential such as food should not be cut off even if the person is working. They will start to make the food stamps less and if it gets to a certain income, you will not get them at all (#90, Arabic, Female).  Food became a problem when I was not working because I was sick (#44, Kinyarwanda, Female).  Honestly this past month, I was very limited in what I could do. It wasn’t enough because the oil here is expensive, ghee is expensive, meat is expensive especially since we have to get halal. I take 290 for food stamps and it’s not enough for me. I buy halal meats and it’s just not enough for me. I worry at the 20th of the month that the food I have is not going to last. So, I worry from the 20th until the day my EBT is recharged for me to use (#83, Arabic, Male) |
| Awareness | This theme refers to informational barriers such as limited knowledge about the U.S. food environment and food programs including their eligibility criteria and application process | Our caseworker mentioned that there are various food assistance programs, but they didn’t provide us with addresses or details; they only said that such help exists (#18, Dari, Male).  I thought the only help you can get in the states was food stamps. So when you mentioned all these things [food pantries] I was surprised. I did not even know about them. (#84, Arabic, Female) |
| Accommodation | This theme refers to whether the providers can meet refugees’ needs and can include translation services, technology, or provision of cultural foods | IRC did give us a computer, but I don’t know how to use it. The computer’s mouse does not work. (Interviewer: do you think having a computer would be helpful?) Yes, then you can do everything on the computer instead of me going there and back. But then again you have to have knowledge on how to use a computer which I do not have (#90, Arabic, Female).  I told them I wished to learn English and they said to me that I needed to buy [a course] to learn. I don’t have any money, I spent all my money the 10 years I spent in Turkey. We came here with nothing to our name. So, when she told me I had to buy, I gave up (#84, Arabic, Female).  For the first year or two I always had a translator with me when I went to appointments. We cannot deny that IRC helped us with a lot of things but a translator was the most useful to me (#87, Arabic, Male).  When we went to the store, we were surprised. They have some stuff that we used to have back home (#50, English, Male).  At first, when we just reached here, we started eating American foods, and it didn’t go well with us, because we were not used to it, and we did not know where to buy African food. But as time goes by, we get used. We also started getting to know where to buy the food we want Most stores we go to have our cultural food. Walmart, Smith, and Costco have the vegetables we like. We go to Farmers Market sometimes to buy different African vegetables. Our friends told us about it (#44, Kinyarwanda, Female).  Farmers Market are great because they have all the vegetables. There are some vegetables we never thought we would eat again in America, but we found them all here (#46, Kinyarwanda, Female).  It’s a friend who told me. When we were talking just like this about food, and she told me that there’s a place where they give free food, and I went there. Only that it’s not food which children like, but if you don’t have anything to eat, at least you can get what to cook for dinner. But I don’t go there a lot because my kids don’t like them. (#47, Kinyarwanda, Female) |
| Acceptability | This theme refers to refugees’ attitudes and comfort with the services and service providers. It can include discrimination and stigma | After all these years, we have gradually started to adapt to life in America and connect with its people. When we first arrived, we were far away from our families, didn’t speak the language, and felt quite isolated. We didn’t have a car, and we didn’t know anyone. Everything was difficult, even the way we dressed. However, over time, we learned and adjusted. Now, we see ourselves integrating with the community and finding solutions to our challenges. It’s a process, but with each passing day, we are becoming more accustomed to our new environment (#19, Dari, Female).  We are cowards and do not like asking for food. We are not used to asking for help. Our personalities don’t allow us to prove that we are hungry or that we are in need of food (#81, Arabic, Female). |
| Availability | This theme refers to sufficient services and resources to meet refugees’ needs, including availability of food resources in their neighborhood (e.g., Farmers Market) and staff availability and capacity to assist refugees with their unmet food needs | This Farmers Market only operates in the summer. It closes during winter. But we can buy more vegetables and refrigerate them and use them later (#33, Kinyarwanda, Female). |
| Accessibility | This theme refers to whether refugees can access food. Barriers and facilitators may include travel time to food resources, access to a vehicle or public transportation, cooking appliances, and cooking skills. | When we arrived in the United States, it was very difficult. We didn’t know the language. Had no friends, and didn’t have a car (#19, Dari, Female).  Many times, things that prohibit us, is that you won’t know where the office is, you don’t know the language, you don’t have a driver’s license to drive to the office. I find these things a problem (#43, Kinyarwanda, Female).  I have to take two buses there and two buses back. So it takes me about 4 hours to get groceries and come home. The wait for the bus takes the longest (#81, Arabic, Female).  Of course, I know how to cook. Everyone in this household cooks. I have YouTube to use when I want to learn how to cook something new (#80, Arabic, Male).  We also make everything at home because first of all it is cheaper for us and also it is healthier (#89, Arabic, Female).  IRC gave us pots and pans but I bought some too. I use the ones I got more because they did give us pots and pans but they aren’t enough for us (#81, Arabic, Female).  They gave us small pots and pans. So, I have to cook twice to make enough food for the family. They are super small. I went to Walmart to get pots and pans (#84, Arabic, Female).  There are some foods that we don’t know how to cook, like fried rice. We know how to cook African food (#45, Kinyarwanda, Female). |
| Themes on temporal patterns of food insecurity | | |
| Finding the first job | As soon as refugees had found a job, they lost all or some part of their SNAP benefits, even though they were still unable to afford their household expenses. The theme directly refers to the Affordability issues | N/A |
| SNAP application renewal | Most SNAP applications must be renewed every 6 months or every time there is a change in household conditions such as income or family size. However, refugees are not familiar with these requirements and often fail to submit their application correctly or in time. This theme refers to the Awareness, Availability, and Accommodation issues | But they cut it off for two months, imagine how that was. We went and try to get our case open again and they told us we had to go and open our case again on our own. We had to give them new paper work for when my husband started working. We did not know any of this information. By the time we were giving them our paperwork and working on our case, they had cut us off of food stamps for two months (#81, Arabic, Female).  After a few months, we did not know what it is, but they closed food stamp, and we spent two months without eating. My husband went to refugee center, and they helped him to reapply, then told us to wait for it in the following month. That’s how we were able to find it again. Things that are hard for us, we go to refugee center. But I cannot say that we know how to re-apply for it on own (#33, Kinyarwanda, Female). |
| Losing caseworkers’ support | Refugees rely on caseworkers’ support to access resources. However, high turnover among caseworkers and the limited time they have to assist refugees (both in terms of high demands and the time period during which caseworker support is available) can reduce refugees’ access to resources. This theme refers to the Availability and Accommodation issues | She was with us for three months then someone else took our case and he was not a good caseworker. He made us lose a lot of programs. We had to ask a friend to help us apply. My husband and I got COVID two months after the new caseworker got our paperwork. We were in need for help for rent. He made us lose the housing program that helped us with bills. We had to pay for our bills ourselves. Even the green card has been late, it has been three years and we still haven’t gotten it. He did not give them my daughter’s health paperwork so we had to go a fix all of that (#89, Arabic, Female).  The first stamp was given to me by the first caseworker. I have had two caseworkers. We were with the first caseworker for four months, but the second caseworker, he hasn’t even reached at my house (#40, Kinyarwanda, Male). |
| Employment fluctuations and household expenditures | Refugees do not always find stable jobs and may face unemployment frequently. Due to limited access to their caseworkers, specifically when they are no longer supported by resettlement agencies, securing employment after a job loss is a significant barrier. In addition, expected and unexpected costs, like those from healthcare, can limit their budget for food. This theme refers to the Affordability issues and can be exacerbated by the Availability and Awareness issues | I don’t have any government assistance from Medicaid or food stamp. This was beyond me because I was sick and could not go back to work that they will accept. I was also not getting treated that at that point I will get better and go back to work because I did not have Medicaid. I accepted my circumstances and decided to start looking for assistance so I can get health. Because when I used to try to go back to work, the employer used to say that, no you can’t work because you don’t have good health, and when I talked to my doctor to write me a note allowing me to go back to work, the doctor said no, I can’t write you a note to go back to work when I haven’t treated you. I was left with no option of what to do. But I can tell you that I suffered a lot during those months. Because of suffering so much, I decided to stop worrying about going back to work and decided to focus on my health. I planned to start going back to the government offices to start looking for assistance. I did all I could to find assistance since I could not go back to work. Food became a problem when I seated, not working because I was sick. When I was working, my husband I worked and put money together to pay bills and buy food. Even though things were sometimes hard especially when it’s around the time of paying bills, I can say that we still managed to get enough food. However, when I had to sit home not working and without food stamp, food became a problem (#44, Kinyarwanda, Female).  My husband was unemployed for nine months, and our food stamps were cut off several times, including once six months. We had to sign papers, but we didn’t manage to do that. We experienced all these difficulties (#16, Dari, Male).  Like now, if a child gets sick, I don’t have a way they can get treated. A child can even end up dying due to not getting treatment. Like now, I pay all the bills, buy clothes and everything and they also need food. You see, in all those things, at least they can help us get a way for the children to get treatment. Because all children don’t have Medicaid. They can help us at least with the children Medicaid and we can focus on finding food for them. Or at least they can help them with food stamp and we can focus on helping them with other things (#47, Kinyarwanda, Female). |
| Themes on suggested strategies by refugees | | |
| Addressing language barriers |  | The people who are new need to know English or have translations. It is better for translations to be available. They can explain food stamps to them and tell them that this is what they have for the month. The caseworker needs to tell the entire family, not just the head of the family, that this is the amount they have for the month (#87, Arabic, Male).  Like now, I don’t speak English. If they can teach me English, I can know where to be going when I need help, who to ask when I need something like looking for a job, then I can also find myself something to eat (#33, Kinyarwanda, Female). |
| Providing a champion |  | Many Afghan immigrants are not familiar with the systems here and may not know what to do. It would be ideal if someone could be sent once a month to check on things. That person could conduct a thorough assessment, looking into everything and helping to advance our situation. They could ask about the workload, and how much income someone can earn in a week and provide guidance accordingly. Personally, my English is good, I can manage my issues, but everything here is documented, and it can be difficult to comprehend. Some important papers come, and it’s hard to understand them, and we can’t take them to anyone for help (#19, Dari, Female).  Personally, I think it would be great if they can continue to guide us on how to do things. Because if I had guidance when I was reapplying for food stamp, it would have helped. If they had at least even prepared me and told me that food stamp will be closed, would be good. The thing of just closing all the benefits; I just came from Africa and suddenly, they close all benefits, it’s not good (#40, Kinyarwanda, Male). |
| Providing information on how to address their unmet needs |  | IRC has to communicate with others that there are places for them to get food. More promotions to help those who need it. Just making sure that information reaches the people that need it (#81, Arabic, Female).  Honestly when the refugee first gets here, they have to explain to them the amount they have. Take them to grocery stores that accept food stamps. Tell them what foods are accepted. They have to give them information. They should have someone designated with them for a month to help them buy things, take them to places. Just to learn how to use food stamps. I felt a lack of knowledge when it came to this situation (#94, Arabic, Female).  Everyone who comes here must receive help. In my opinion, someone must teach them everything, from how to navigate the markets to what types of food are available and what is not. They should also learn how to obtain household items and how to use banking cards. This support is vital because many people face significant challenges. I have seen and experienced how difficult it can be. My own experience has shown me just how important it is to have guidance in these areas (#19, Dari, Female).  They can teach me how to do those applications for benefits, because it’s the one I don’t know. Because our caseworker left us in the early months. There are many things we don’t know (#40, Kinyarwanda, Male). |
| Extending or expanding SNAP benefits |  | They need to increase the minimum amount in order to qualify for food stamps and stuff. For example, they need to check how much that persons is paying for the mortgage. Yes, the income may be too high but there are other stuff that person needs to cover. If you look at how much I make a month, yes that’s okay. But I’m spending all that even end up going in the negative in the account (#50, English, Male).  There are people who like to leave the food assistance programs to work and grow themselves. But those who have kids should still have food stamps unless there are four people who work. If that was the case, then it would be enough for them. But other than that, they would not be able to afford things (#88, Arabic, Female). |
| Providing gardens to grow food |  | I think everyone can get a garden. Even if you have a job, you can still have a garden and farm for yourself like vegetables (#34, Kinyarwanda, Female). |
