# Supplementary material for: Addressing food insecurity among U.S. refugees, considering the temporal patterns of food insecurity after resettlement: Qualitative insights from Utah
Source: PLoS One. 2025 Jul 17;20(7):e0327645. doi: 10.1371/journal.pone.0327645 (PMC12270138; doi:10.1371/journal.pone.0327645)
Supplement: Attachment — Submitted filename: Response to Reviewers.docx [file pone.0327645.s004.docx]

Thank you for the opportunity to revise and resubmit our manuscript (PONE-D-25-21762), entitled “Addressing food insecurity among U.S. refugees, considering the temporal patterns of food insecurity after resettlement: Qualitative insights from Utah” for reconsideration by *PLOS ONE*.

We thank the editor and reviewers for their useful comments. We appreciate the opportunity to address the comments by submitting a revision. We have considered all comments and believe that the revisions have addressed these comments and greatly improved the paper. Please note the change in the title in response to Reviewer 1’s comment #1.

Below, we have addressed each reviewer’s concerns by explaining in detail the responses (**in** **bold**) to their questions/concerns (numbered). We also refer the reviewers to the line number within the revised manuscript.

# Editor

1. Please ensure that your manuscript meets PLOS ONE's style requirements, including those for file naming. The PLOS ONE style templates can be found at

<https://journals.plos.org/plosone/s/file?id=wjVg/PLOSOne_formatting_sample_main_body.pdf> and

[https://journals.plos.org/plosone/s/file?id=ba62/PLOSOne_formatting_sample_title_authors_affiliations.pdf](https://track.editorialmanager.com/CL0/https:%2F%2Fjournals.plos.org%2Fplosone%2Fs%2Ffile%3Fid=ba62%2FPLOSOne_formatting_sample_title_authors_affiliations.pdf/1/010f019740c12c20-3052b622-5bec-4711-b673-984b278bcda8-000000/4GVOubocY5f9mIKLRZXSrSJntvXFzTN4Dcer7rOmSeM=214)

**We revised the file names and adhered to other style requirements.**

2. In the ethics statement in the Methods, you have specified that oral consent was obtained. Please provide additional details regarding how this consent was documented and witnessed, and state whether this was approved by the IRB.

**Oral consent was recorded and transcribed. This procedure was approved by the IRB. We added these clarifications in the methods section.**

3. Thank you for stating the following financial disclosure:
   “We received funding from the University of Utah’s School of Medicine: FY24 Vice President for Research Incentive Seed Grant Competition”. Please state what role the funders took in the study. If the funders had no role, please state: "The funders had no role in study design, data collection and analysis, decision to publish, or preparation of the manuscript."
   If this statement is not correct you must amend it as needed. Please include this amended Role of Funder statement in your cover letter; we will change the online submission form on your behalf.

**We added the following statement in the funding statement (see next comment) and the cover letter: The funders had no role in study design, data collection and analysis, decision to publish, or preparation of the manuscript.**

4. Thank you for stating the following in the Acknowledgments Section of your manuscript:
   “Funding: We received funding from the University of Utah’s School of Medicine: FY24 Vice President for Research Incentive Seed Grant Competition. Interviewees: We truly appreciate our part-time staff who conducted the interviews. Thank you, Lin Alsubhi, Juliet Munyambanza, and Mahsa Qaderi.” We note that you have provided funding information that is not currently declared in your Funding Statement. However, funding information should not appear in the Acknowledgments section or other areas of your manuscript. We will only publish funding information present in the Funding Statement section of the online submission form. Please remove any funding-related text from the manuscript and let us know how you would like to update your Funding Statement. Currently, your Funding Statement reads as follows:
   “We received funding from the University of Utah’s School of Medicine: FY24 Vice President for Research Incentive Seed Grant Competition”
   Please include your amended statements within your cover letter; we will change the online submission form on your behalf.

**We removed the funding information from the Acknowledgement section.**

**We also included this statement in the cover letter so that you can update the funding statement accordingly: We received funding from the University of Utah’s School of Medicine: FY24 Vice President for Research Incentive Seed Grant Competition. The funders had no role in study design, data collection and analysis, decision to publish, or preparation of the manuscript.**

5. In the online submission form, you indicated that “De-identified transcripts can be shared upon signing an agreement prior to release.” All PLOS journals now require all data underlying the findings described in their manuscript to be freely available to other researchers, either 1. In a public repository, 2. Within the manuscript itself, or 3. Uploaded as supplementary information. This policy applies to all data except where public deposition would breach compliance with the protocol approved by your research ethics board. If your data cannot be made publicly available for ethical or legal reasons (e.g., public availability would compromise patient privacy), please explain your reasons on resubmission and your exemption request will be escalated for approval.

**De-identified transcripts are now available in a public repository**: <https://figshare.com/articles/dataset/De-identified_English_Transcripts_of_36_interviews/29318549>

6. Please include captions for your Supporting Information files at the end of your manuscript, and update any in-text citations to match accordingly. Please see our Supporting Information guidelines for more information: <http://journals.plos.org/plosone/s/supporting-information>.

**Captions for supporting information are now provided after the references. The in-text citations were also updated.**

7. Please review your reference list to ensure that it is complete and correct. If you have cited papers that have been retracted, please include the rationale for doing so in the manuscript text, or remove these references and replace them with relevant current references. Any changes to the reference list should be mentioned in the rebuttal letter that accompanies your revised manuscript. If you need to cite a retracted article, indicate the article’s retracted status in the References list and also include a citation and full reference for the retraction notice.

**We reviewed the references and all of them are current and complete. We did not add new references.**

8. While revising your submission, please upload your figure files to the Preflight Analysis and Conversion Engine (PACE) digital diagnostic tool, [https://pacev2.apexcovantage.com/](https://track.editorialmanager.com/CL0/https:%2F%2Fpacev2.apexcovantage.com%2F/1/010f019740c12c20-3052b622-5bec-4711-b673-984b278bcda8-000000/ciyDX2eWJICOZYSO3eWOYU95unLW8CWeHjSQErdixUM=214). PACE helps ensure that figures meet PLOS requirements. To use PACE, you must first register as a user. Registration is free. Then, login and navigate to the UPLOAD tab, where you will find detailed instructions on how to use the tool. If you encounter any issues or have any questions when using PACE, please email PLOS at [figures@plos.org](mailto:figures@plos.org). Please note that Supporting Information files do not need this step.

**Our manuscript does not contain any figures.**

# Reviewer #1

1. Title: The current title may give the impression that this is a review article, when in fact it reports original research. To better reflect the nature of the study, it is recommended to specify the study design—for example, by indicating that qualitative methods were used. This will help clarify the article's methodological approach and contribution for potential readers. Additionally, the title could be more focused by incorporating key components of the findings, such as the circumstances under which refugees are most vulnerable and their preferred strategies for addressing food insecurity.

**Thank you for your suggestion. We revised the title to:** **Addressing food insecurity among U.S. refugees, considering the temporal patterns of food insecurity after resettlement: Qualitative insights from Utah.**

2. Abstract: [Line 26-27] The clear identification of the research gap is a strong aspect of the study. Clearly stating the objective of the study would enhance the clarity and focus of the manuscript.

**We added the objectives in the abstract: The objectives were to explore FI experiences among refugees to identify time points at which accessing food becomes a problem and to identify refugees’ preferred strategies to address FI.**

3. Abstract: [Line 28] Including the sampling method would be a valuable addition. It would also be helpful to briefly describe the process of refugee recruitment.

**We revised the Methods section of the Abstract to read as “In collaboration with one of the U.S. resettlement agencies in Utah, refugees were recruited for semi-structured interviews using convenience and snowball sampling”.**

4. Introduction: [Additional suggestion] The content is well-structured and informative. However, providing additional information on what refugees are permitted or restricted from doing upon arrival in the U.S. would help enlighten international readers and offer a more comprehensive understanding of their resettlement experience.

**We added a sentence at the beginning of Paragraph two of the Introduction section, which reads as “The U.S. refugees are authorized to work in the U.S., can access public benefits, and will receive resettlement services.” However, there is no food-related restriction to mention.**

5. Methods: [line 105] How about the duration of arriving at U.S.? No exclusion? How about if the refugee come alone? Refugee from food insecure household?

**We had no inclusion criteria on the length of U.S. residency or food insecurity status, as we believed that all refugees could provide insight into food access issues as long as they were adults and could do the interview in the selected languages.**

6. Methods: [line 116] Any permission needed for conducting the study at the resettlement centers? The information might be beneficial the future studies which are planned to conduct data collection at the resettlement centers.

**No permission is required as long as the IRB approves the project. However, recruitment and conducting interviews with refugees is easier if researchers can partner and collaborate with resettlement agencies.**

7. Methods: [line 116] Could you clarify how the study determined that the number of interviews conducted was sufficient to capture comprehensive and meaningful insights?

**We initially started with 36 interviews. After conducting all these interviews and analyzing the transcripts, we used thematic saturation to decide whether further interviews are needed. We found that saturation was reached, and therefore, we did not conduct any further interviews. This is mentioned in the Methods section.**

8. Methods: [line 120] It is recommended to insert a subheading titled 'Instruments' to clearly present the tools or materials used for data collection.

**We added the subheading. Thank you for the suggestion.**

9. Methods: [Additional suggestion] It would be helpful to include any steps taken to ensure the quality and trustworthiness of the qualitative data. Additionally, providing information about the interviewers—such as their training and experience in conducting qualitative research—would strengthen the credibility of the study.

**We amended the Data Collection section in the Methods accordingly. Our staff members were provided with up to 20 hours of training on Human Subject Research and conducting interviews. They were also given guidance on how to translate and transcribe the interviews.**

10. Results: [line 195] Are there any participant quotes related to 'cost of food' and 'the inability to afford food' provided in the supplementary file? These quotes are not presented in the main text.

**Yes, they are included in Appendix B.**

11. Results: [line 200 & 274] It is recommended to remove the company names to protect privacy and ensure confidentiality for the organizations involved.

**We removed the company names.**

12. Results: [line 358] It would be helpful to provide an explanation of the term 'champion,' especially since it may not be familiar to international readers.

**We added this definition to that sentence: someone who supports a cause or person.**

13. Discussion: [line 409] It may be beneficial to categorize the themes in the results section into 'income-related' and 'non-income-related' barriers. This would provide a clearer reflection of the distinct challenges faced by refugees.

**We categorized barriers in the “themes relating to FI experiences” section into income- and non-income-related barriers.**

14. Discussion: [Additional suggestion] How have other countries addressed similar issues? Are there any solutions or best practices from other countries that could be applied or learned from in addressing these challenges?

**Answering this question requires a comprehensive literature review on those interventions used in other countries, which is the next step of our research.**

15. Conclusion: [Additional suggestion] What is the potential feasibility of implementing the suggested methods in addressing food insecurity (FI)?

**This is beyond the scope of the manuscript, as it requires having the resettlement agencies rate the perceived effectiveness, feasibility, and priority of those interventions, considering their organizational constraints. However, the insights provided here can initiate the discussion with relevant organizations to think about and identify potential solutions for refugees.**

# Reviewer #2

1. The methodology should be more clearly stated.

**We amended the Methods section in response to Reviewer 1’s comments #5-9.**

2. The result should be presented with some graphs, not only tables.

**This is not a requirement of the journal, and we believe that the tables provided in the Results section adequately reflect the findings of our research.**

3. The strengths and limitations should be clearly stated.

**Please refer to the Limitations section. The discussion section also starts with highlighting the strength of this paper.**

4. All through English, grammar correction is needed.

**Thank you for the comment. We proofread the manuscript again.**
